# Oral green tea catechins do not provide photoprotection from direct DNA damage induced by higher dose solar simulated radiation: A randomized controlled trial

**DOI:** 10.1016/j.jaad.2017.08.021

**Published:** 2018-02

**Authors:** Mark D. Farrar, Raqib Huq, Sarah Mason, Anna Nicolaou, Kayleigh A. Clarke, Tristan P. Dew, Gary Williamson, Rachel E.B. Watson, Lesley E. Rhodes

**Affiliations:** a Centre for Dermatology, Division of Musculoskeletal and Dermatological Sciences, School of Biological Sciences, Faculty of Biology, Medicine and Health, The University of Manchester, Manchester Academic Health Science Centre, Salford Royal NHS Foundation Trust, Manchester, United Kingdom; b Division of Pharmacy and Optometry, School of Health Sciences, Faculty of Biology, Medicine and Health, The University of Manchester, Manchester Academic Health Science Centre, Manchester, United Kingdom; c School of Food Science and Nutrition, University of Leeds, Leeds, United Kingdom; d Department of Health Professions, Faculty of Health, Psychology and Social Care, Manchester Metropolitan University, Manchester, United Kingdom

*To the Editor:* Exposure to ultraviolet radiation (UVR) in sunlight is the principal cause of most skin cancers. Despite topical sunscreen availability, skin cancer incidence continues to rise with substantial financial burden to health care. Systemic photoprotection through safe dietary means has gained interest. Green tea catechins (GTC) administered topically or orally are chemopreventive in mouse models of UVR-induced skin cancer, potentially through reduced DNA damage or enhanced DNA repair.^1^ Topical GTC protected against UVR-induced cyclobutane pyrimidine dimers (CPD) in human skin,^2^ but no information exists regarding the impact of oral GTC in humans.

We performed a double-blind, randomized, placebo-controlled trial in healthy white adults (13 male and 37 female; 18-65 years of age; Fitzpatrick skin phototypes I and II) who received 1080 mg GTC (equivalent to 5 cups/day) with 100 mg vitamin C (n = 25) or placebo maltodextrin (n = 25) daily for 12 weeks.^3^ Vitamin C was used as previously^3^ to stabilize GTC in the gut lumen.^4^ A high dose proinflammatory (3 × minimal erythema dose [MED]) challenge with solar simulated UVR (5% UVB, 95% UVA) was applied to buttock skin pre- and postsupplementation. Immunohistochemical staining with CPD-positive cell quantification was performed in skin samples taken 24 hours after UVR exposure. In a further, before-after time-course pilot study, 5 subjects (2 male and 3 female; 21-31 years of age, Fitzpatrick skin phototype II) received GTC with vitamin C for 4 weeks with UVR-induced (2MED) CPD assessed pre- and postsupplementation. Urinary epigallocatechin glucuronide analysis assessed compliance.^3^ CPD-positive cells/1000 μm^2^ in UVR-irradiated epidermis postsupplementation was compared between active and placebo groups by analysis of covariance with baseline data as the covariate.

**Fig 1:**
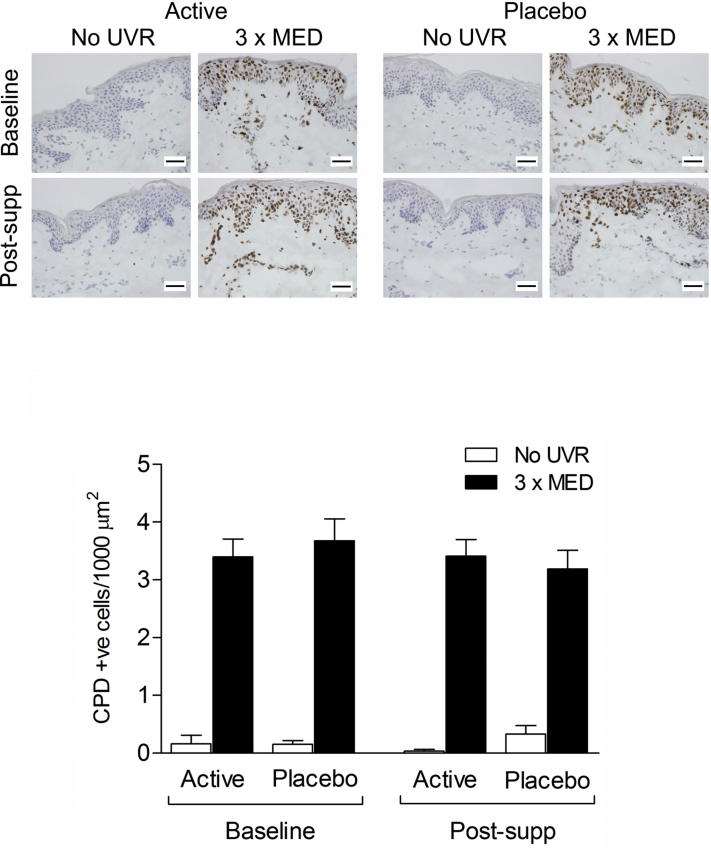
Ultraviolet radiation (UVR)-induced cyclobutane pyrimidine dimers (CPD) in skin from a randomized, controlled study of 12 weeks of supplementation with green tea catechins and vitamin C versus placebo. Representative immunohistochemistry and quantification of CPD in UVR-exposed (3 × minimal erythema dose; MED) and unexposed skin at baseline and postsupplementation. Active n = 20 subjects, placebo n = 24 subjects. Scale bar = 50 μm.

**Fig 2:**
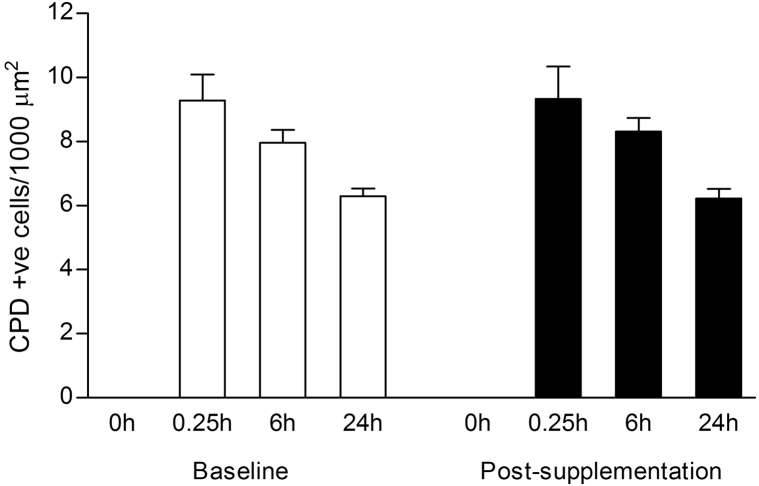
Time course of ultraviolet radiation–induced cyclobutane pyrimidine dimers (CPD) in skin at baseline and after 4 weeks supplementation with green tea catechins and vitamin C. Data are mean + SD number of CPD-positive cells/1000 μm^2^ epidermis in unexposed skin (0 hours) and at 0.25, 6, and 24 hours after UVR exposure of skin. n = 5 subjects.

UVR induced CPD-positive cells in the epidermis and to a much lesser extent the dermis (Fig 1). Following the 12-week intervention, there was no difference between active and placebo groups in number of CPD-positive cells in UVR-irradiated epidermis at 24 hours (*P* = .81; Fig 1), the active group showing mean (SD) 3.4 (1.3) cells/1000 μm^2^ at both baseline and postsupplementation (*P* = .74), and placebo 3.7 (1.8) and 3.2 (1.5) cells/1000 μm^2^ at baseline and postsupplementation, respectively (*P* = .20). Evaluation of a moderate (2MED) UVR dose and further time points similarly found no effect of supplement on CPD (Fig 2).

Our findings contrast with oral studies in mice^1^ and topical human studies which found that GTC protected against direct DNA damage.^2^ Oral green tea consumption is difficult to control in mice, and topical human studies potentially have higher local skin concentrations of GTC than those after ingestion. We previously showed GTC and metabolites are bioavailable in human skin but with intersubject variability in both the range of specific metabolites and concentrations detected,^5^ and further studies may examine means to enhance skin uptake/bioavailability. Strengths include the robust study design, tablet count and biochemically determined subject compliance, and use of solar-simulating UVR giving more relevance to natural sun exposure than studies using primarily UVB radiation.^1^, ^2^ Our study scope is limited to erythemal UVR and direct DNA damage; we demonstrate that oral GTC is no substitute for topical sunscreen but have not excluded an adjunctive role to sunscreen. Protection against low-dose UVR exposure as in everyday life, and against oxidative DNA damage, warrants future study.
